# Schwannoma of the lateral nasal wall: A case report

**DOI:** 10.1002/ccr3.5451

**Published:** 2022-02-15

**Authors:** Behrouz Barati, Malihe Mohseni, Mahboobe Asadi, Forogh Mangeli

**Affiliations:** ^1^ Otolaryngology Department Shahid Beheshti University of Medical Sciences Tehran Iran; ^2^ Pathology Department Shahid Beheshti University of Medical Sciences Tehran Iran

**Keywords:** benign nerve sheath tumor, nasal cavity, schwannoma

## Abstract

Nasal schwannomas account for about 4% of head and neck schwannomas. We report a rare case of lateral nasal wall schwannoma presenting as a nasal mass in a 70‐year‐old man.

## Graphical abstract

Nasal cavity schwannoma is rare, and a definite diagnosis is made only with the histopathological report.

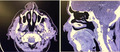

## 1 INTRODUCTION

Schwannomas are a group of tumors of the peripheral nervous system (PNS). In fact, they are an abnormal and usually benign proliferation of Schwann cells (cells involved in the formation of the myelin sheath in the PNS). Hence, any nerve in the PNS that contains a myelin sheath can be the origin of schwannoma, so this type of neoplasm can occur anywhere in the body.^1^ It has been reported that in the head and neck region, the most common site of schwannoma is the cerebellopontine angle (vestibular schwannoma).^2^ Nevertheless, many articles have reported the development of schwannoma in the pharynx, oral cavity, parotid, larynx, and scalp.^3^ About 4% of head and neck schwannomas arises from the sinonasal region. Several studies reported schwannomas of the medial wall of the nasal cavity (septal schwannomas).^4^ In this manuscript, we report a very rare schwannoma originating from the lateral nasal wall.

## 2 CASE REPORT

**FIGURE 1:**
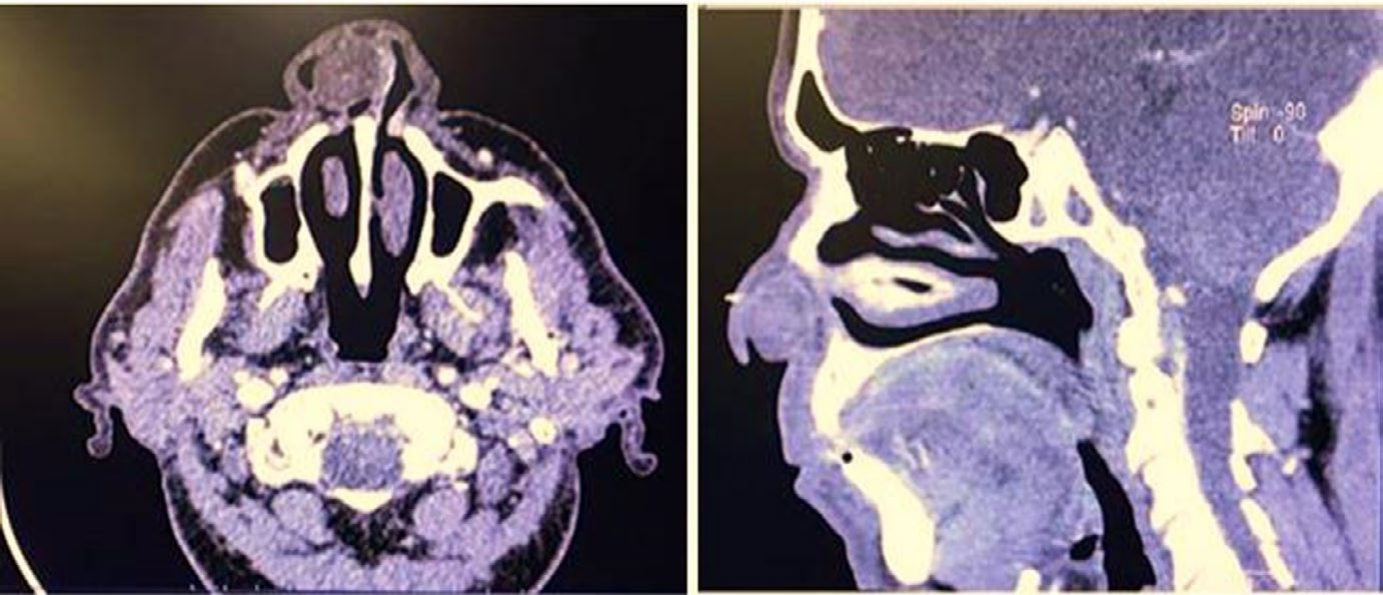
Contrast‐enhanced computed tomography showed a well‐defined hypodense lesion in the anterior part of the right nasal cavity

**FIGURE 2:**
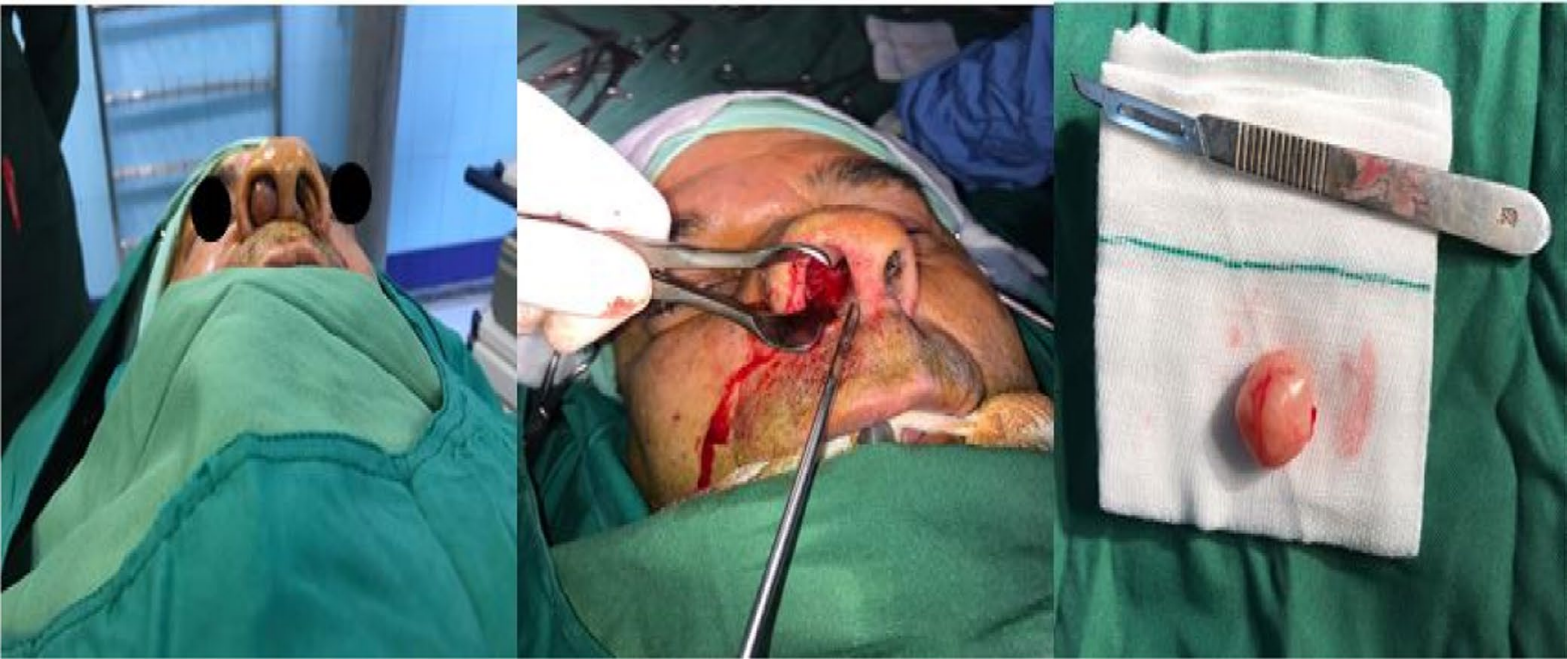
Endonasal excision of the right nasal cavity and the mass was removed completely

**FIGURE 3:**
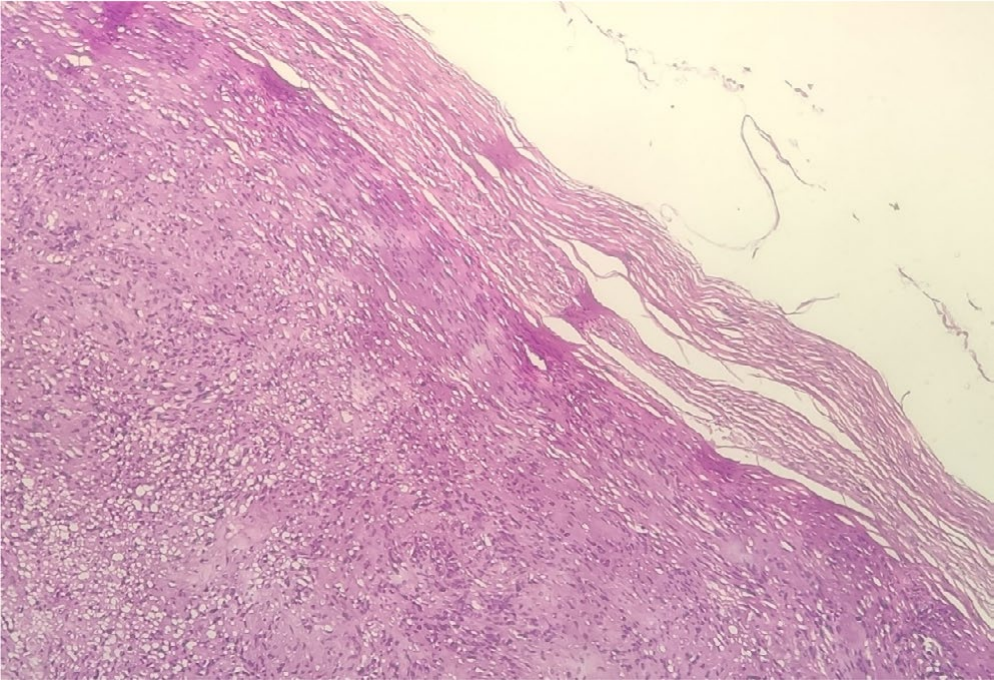
Spindle cell neoplasm with thick capsule (H&E stained ×10 slide)

**FIGURE 4:**
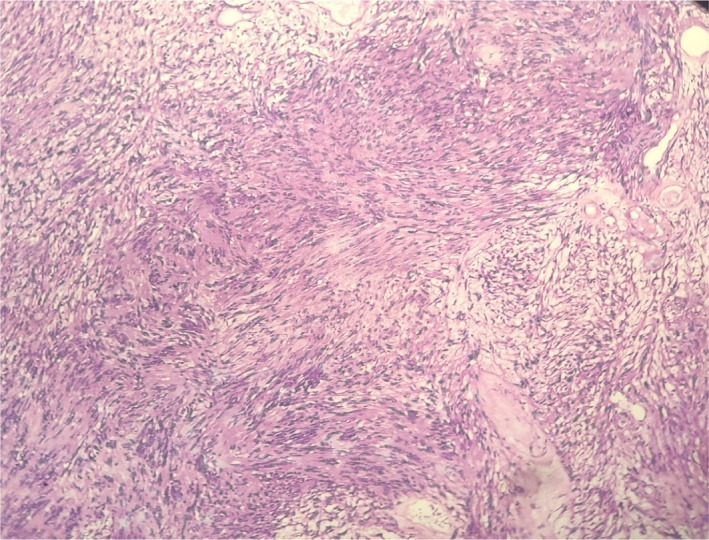
Antoni A (compact hypercellular) and Antoni B (and myxoid hypocellular) areas. (H&E stained ×10 slide)

A 70‐year‐old man with unilateral progressive nasal obstruction that started about 2 years ago referred to the otolaryngology department of Taleghani Hospital, Shahid Beheshti University of Medical Sciences, Tehran, Iran. The patient was employed in the textile industry. There was no positive history of nasal trauma, smell disorders, posterior nasal discharge, epistaxis, chronic cough, rhinorrhea, sneezing, headache, diplopia, and facial pain. His past history included a known subject of type 2 diabetes mellitus. Furthermore, his family history was not remarkable. On nasal endoscopy, there was a space‐occupying mass in the right nasal vestibule that prevented further observation. The remaining ear, larynx, pharynx, head, and neck examinations were normal. Contrast‐enhanced computed tomography showed a soft tissue mass (40–45 HU) with mild heterogeneous enhancement in the right nasal cavity with no destructive effect on adjacent structures (Figure 1). Endonasal excision of the right nasal cavity mass was done under general anesthesia. Intraoperative observation revealed a pedunculated skin‐covered mass originated from the lateral nasal wall. The mass was totally excised, and its pedicle was cauterized (Figure 2). Homeostasis was achieved, and the right nasal cavity was packed with ribbon gauze for 24 h. The postoperative was uneventful. The microscopic findings of the resected mass lesion show a biphasic neoplasm with a well‐defined border and thick capsule (Figure 3), composed of spindle cells with narrow, elongated, and wavy nuclei with tapered ends, dense chromatin, and ill‐defined cytoplasm arranged in compact hypercellular (Antoni A) areas and myxoid hypocellular (Antoni B) areas (Figure 4) with the presence of nuclear palisading, Verocay bodies (figure the pathology report was compatible with schwannoma.

## 3 DISCUSSION

Schwannomas are benign slow‐growing masses with a less destructive pattern.^5^ Obstruction of the nasal cavity is the most common clinical presentation in nasal cavity schwannoma, although other less common symptoms include visual disorders, facial nerve palsy, facial pain, and exophthalmos.^6^ In the head and neck region, neck soft tissue, parapharyngeal space, and oropharynx are the most commonly diagnosed locations. Hence, schwannomas limited to the nasal cavity are extremely rare, with approximately 20 reported cases in the literature.^7^ In most reports, the nasal cavity schwannoma originated from the nasal septum,^6^, ^7^, ^8^, ^9^ and only in one case, the tumor origin was reported to be from the nasal sidewall.

This disorder usually occurs in the fifth and sixth decades of life, and issues related to sex and race do not play a significant role in its occurrence.^10^ It has been reported that nasal cavity schwannomas originate from the maxillary and ophthalmic nerves, sphenopalatine ganglion, and other nerves that innervated the nasal mucosa.^8^ In the present patient, nasal cavity schwannoma originated from the lateral nasal wall, and given that the lateral wall is innervated by anterior ethmoidal nerve, lateral posterior inferior nasal, and lateral posterior superior nasal, it seems that aforementioned schwannoma is associated with above nerve; however, due to the complex pathomechanism of schwannoma, it is not possible to make a definite statement about the origin of this type of schwannoma and the issue is open to as previously mentioned, the pathomechanism of schwannoma is very complex and several factors are involved in its development. It has been suggested that oxidative stress plays a crucial role in the pathogenesis of schwannoma.^11^ Based on the review of literature, in some cases of nasal schwannoma, the patient had diabetes mellitus.^8^ In our study, the patient had diabetes for 4 years. Although diabetes mellitus increases free radicals through lipotoxicity and glucotoxicity,^12^ we cannot say certainty that diabetes has been one of the risk factors for schwannoma in our patients, and this requires precise cellular and molecular studies. Differential diagnosis between different tumors of the nasal cavity such as squamous cell carcinoma, schwannoma, lymphoma, fibrous dysplasia, chondrosarcoma, and extension of the angiofibroma of the nasopharynx is very difficult based on clinical findings. As we know, medical imaging such as CT scans may not provide valuable findings for differential diagnosis, but they are useful in evaluating the size, diameter, and extent of mass.^13^ The most accurate method for the definitive diagnosis of schwannoma is histopathological assays. Schwannomas generally have a smooth appearance and are covered by a capsule derived from the perineurium of the nerve. Also, these tumors grow eccentrically to the nerve from which they arise. Another diagnostic criterion is the presence of spindle cells with a basophilic histological pattern. In the present study, the pathology report described a benign spindle cell neoplasm that was compatible with schwannoma.^5^ In macroscopic view, schwannoma is lonely, well‐demarcated with a round or oval shape, yellowish to grayish in color, and shiny on cut surface.^6^ In our study, the macroscopic appearance of the mass was in accordance with schwannoma criteria, but as mentioned, it has no high diagnostic value (Figure 2). According to the nature of schwannoma, excision by external approach or endonasal surgery is the treatment of choice based on tumor size and extension.^14^ With this in mind, in our case due to the tumor size and its limited extension, the endonasal approach was selected.

## 4 CONCLUSION

Nasal cavity schwannoma is rare, and definite diagnosis is made only with histopathological reports. It seems that nasal cavity schwannomas originate from trigeminal nerve branches, but the issue is open to discussion. Furthermore, endoscopic surgery is an efficient method to remove most of the benign mass of nasal cavity such as schwannoma.

## CONFLICT OF INTEREST

The authors made no disclosures.

## AUTHOR CONTRIBUTION

MM and MA contributed to the manuscript preparation. BB, MA, and MM contributed to the patient management. FM is pathologist who contributed to the pathological report. All authors read and approved the final manuscript.

## ETHICAL APPROVAL

Because this report involves no experiment, ethics approval is waived.

## CONSENT

Written informed consent was obtained from the patient for publication of this case report and accompanying images. A copy of the written consent is available for review by the Editor‐in‐Chief of this journal on request.

## PATIENT PERSPECTIVE

I felt so astonishing when I saw this huge mass, which was in my nose! I hope that my nose size return to normal.

## Data Availability

The data that support the findings of this study are available on request from the corresponding author [Mahboobe Asadi].
